# The Efficacy of Combined Decompression of Carpal and Cubital Tunnel in the Setting of Cervical Spine Stenosis: A Retrospective Analysis

**DOI:** 10.7759/cureus.73827

**Published:** 2024-11-16

**Authors:** Mutaz Alsaleh, Joshua Abishek, Amr Basha, Hussain Selmi, Mohammed Al-Azzawi, Daoud Makki

**Affiliations:** 1 Trauma and Orthopaedics, Watford General Hospital, West Hertfordshire Teaching Hospitals NHS Trust, Watford, GBR; 2 Trauma and Orthopaedics, NHS Tayside, Dundee, GBR; 3 Trauma and Orthopaedics, West Hertfordshire Teaching Hospitals NHS Trust, Watford, GBR

**Keywords:** carpal tunnel release, carpal tunnel syndome, cervical spine stenosis, cubital neuropathy, cubital tunnel surgery

## Abstract

Background and objective

Hand numbness, often associated with carpal tunnel syndrome (CTS) and cubital tunnel syndrome (CuTS), significantly impacts the quality of life. This study aimed to evaluate the effectiveness of combined carpal and cubital tunnel decompression (CCTD) in patients with concurrent mild to moderate cervical spine stenosis when compared to single decompression procedures.

Methods

We retrospectively reviewed the records of 100 patients who underwent decompression surgery for hand numbness and concurrent cervical spine stenosis, with positive electromyography (EMG) results in some cases, between January 1, 2023, and January 1, 2024. Surgical interventions included carpal tunnel decompression (CTD, n=91), cubital tunnel decompression (CuTD, n=4), and combined procedures (n=5).

Results

Of the 100 patients (aged range: 32-92 years), 11 with coexisting cervical stenosis reported neck pain. Those who underwent CCTD reported significantly lower pain severity and interference scores, as well as better functional outcomes, compared to single decompression groups. Despite the small number of patients (n=5) in the combined decompression group, preliminary findings suggest potential benefits related to this approach.

Conclusions

Simultaneous decompression of the carpal and cubital tunnels may offer superior symptom relief and functional improvement in patients with mild to moderate cervical spine stenosis, highlighting the importance of addressing cervical spine pathology in cases of hand numbness. However, further research with larger sample sizes is warranted to validate these preliminary results and better understand the efficacy of combined decompression for complex cases.

## Introduction

Hand numbness involves a common constellation of symptoms and clinical findings that can be caused by nerve compression syndromes, systemic diseases, and cervical spine pathology, leading to a significant impact on the quality of life [1]. Patients usually present with complaints of tingling, burning, or weakness. Common causes of this condition include carpal tunnel syndrome (CTS), cubital tunnel syndrome (CuTS), and cervical radiculopathy [2-5].

CTS is the most common mononeuropathy condition [6]. Median nerve compression causes symptoms in the thumb, as well as the second, third, and radial half of the fourth digit [6,7]. CuTS, on the other hand, results from ulnar nerve compression at the level of the elbow [8]. Similarly, cervical radiculopathy, stemming from cervical spine stenosis, can also lead to hand numbness, with varying patterns of symptoms depending on the roots affected [5,9]. Predisposing factors can vary, including certain occupations, activities, diabetes, and peripheral neuropathy [5,6,10]. The underlying cause of the patient’s hand numbness is determined through clinical examination, the use of nerve conduction studies (NCS), and MRI if a cervical cause is suspected. Surgical interventions can be considered when conservative measures fail to provide relief.

Since it is quite common for patients to have both cubital and carpal tunnel at the same time [9], the clinical waters are further muddied when cervical causes are thrown into the mix. Cervical radiculopathy in isolation can mimic the symptoms of CTS and CuTS and manifest as a “double crush syndrome,” characterized by concurrent compression of the peripheral nerve at two sites, which has a high prevalence [11-13]. This syndrome often necessitates careful evaluation and tailored treatment strategies, as cervical radiculopathy can exacerbate symptoms of both CTS and CuTS [14-16]. Furthermore, research has shown that double crush syndrome can be assessed and characterized through clinical, radiological, and electrophysiological evaluations, which are critical for an accurate diagnosis and effective management [17,18]. Electrophysiological studies have also been instrumental in exploring the underlying mechanisms of double crush syndrome and its impact on nerve function [19]. Patients with concurrent cervical spine stenosis and hand numbness present a unique challenge in management, and there is a scarcity of evidence guiding successful clinical practice. In this retrospective analysis, we aim to investigate the interplay of carpal tunnel decompression (CTD) and cubital tunnel decompression (CuTD) in symptomatic patients with concurrent cervical spine stenosis.

## Materials and methods

We conducted a retrospective analysis of 100 patients who underwent surgeries for CTD (n=91, 91%) and CuTD (n=4, 4%), with some patients (n=5, 5%) undergoing combined carpal and cubital tunnel decompression (CCTD) between January 2023 and January 2024. The size of the sample was proposed by a statistician to ensure that the error in the conclusion does not exceed 10%. All patients were over the age of 18 years and experienced hand numbness as the presenting complaint. Patients were screened for mild to moderate cervical spine stenosis according to Kang's MRI grading system, and those with severe cervical spine stenosis (grade III and above) were excluded. Additionally, those who had previously undergone cervical spine surgery and those with systemic conditions such as diabetes mellitus, rheumatoid arthritis, or other conditions known to affect nerve function were also excluded.

Postoperative outcomes were assessed using the Brief Pain Inventory (BPI), which includes two primary components: pain severity and pain interference. Pain severity measures the intensity of pain experienced by the patient, rated on a scale from 0 (no pain) to 10 (worst pain imaginable). Pain interference evaluates how much pain disrupts the patient’s daily activities, also rated on a scale from 0 to 10, with higher scores indicating more significant interference [20,21]. We assessed the patients' functional disability postoperatively by using the QuickDASH (Quick Disabilities of the Arm, Shoulder, and Hand), which was also used to measure patients’ perceptions of their physical function and symptoms. The QuickDASH score ranges from 0 to 100, with higher scores representing greater disability. It is widely used to assess functional outcomes in patients undergoing treatment for upper extremity disorders [22].

Descriptive statistics were used to summarize the demographic and clinical characteristics of the study population, including age, sex, and the distribution of surgical procedures. Continuous variables, such as age and outcome scores, were reported as means with standard deviations (SD) or medians with interquartile ranges (IQR), depending on the data distribution. Categorical variables, such as sex and the type of decompression surgery, were presented as frequencies and percentages. Chi-square tests were used to evaluate associations between categorical variables. Statistical significance was set at a p-value <0.05.

## Results

This study included a total of 100 patients, all of whom presented with hand numbness and underwent either CTD (n=91), CuTD (n=4), or CCTD (n=5). The age of the cohort ranged from 32 to 92 years, with a mean age of 62 years. The sample consisted of 35% men (n=35) and 65% women (n=65), with neck pain reported in 11% of the individuals (n=11). Among the patients with cervical spine stenosis, MRI findings indicated that 67% had mild stenosis (n=67) and 33% had moderate stenosis (n=33). Additionally, 11 patients with cervical spine pathology reported neck pain, and electromyography (EMG) findings revealed evidence of cervical radiculopathy in a subset of these patients.

**Figure 1:**
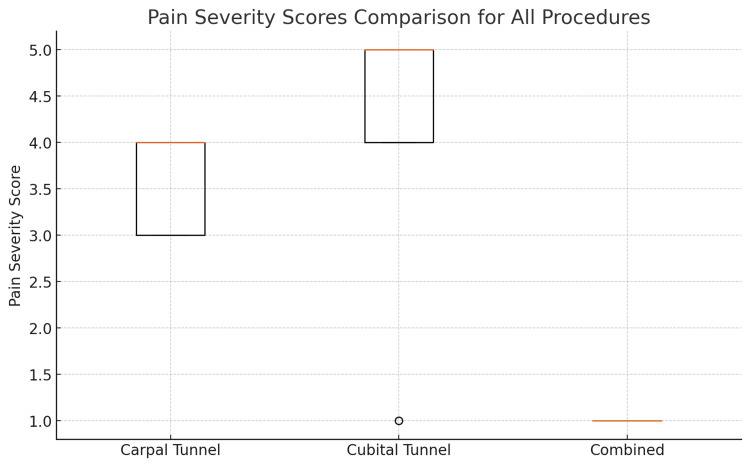
BPI - pain severity This box plot shows a comparison of pain severity scores among patients who underwent carpal tunnel decompression, cubital tunnel decompression, or both procedures together. The box displays the middle range of scores, with the line inside the box marking the median score. Any unusually high or low scores are shown as separate points BPI: Brief Pain Inventory

Regarding pain severity, the median score for patients who underwent CTD was 4/10, while the score for those who received CuTD was slightly higher at 5/10. Patients who underwent CCTD reported a significantly lower median pain severity score of 1/10. Statistical analysis revealed that pain severity scores were significantly lower in the CCTD group compared to the CTD and CuTD groups (p<0.05) (Figure 1) (Table 1).

**Table 1:** Statistical analysis of pain severity, pain interference, and QuickDASH scores across decompression groups Statistical significance was set at a p-value of <0.05. P-values less than 0.05 indicate a statistically significant difference between the compared groups QuickDASH: Quick Disabilities of the Arm, Shoulder, and Hand

Comparison	Values compared	P-value
Pain Severity		
Carpal tunnel vs. cubital tunnel	60 scored 4, 31 scored 3 (carpal tunnel); 3 scored 5, 1 scored 1 (cubital tunnel)	0.047
Carpal tunnel vs. combined decompression	60 scored 4, 31 scored 3 (carpal tunnel); all scored 1 (combined)	0.0000106
Cubital tunnel vs. combined decompression	3 scored 5, 1 scored 1 (cubital tunnel); all scored 1 (combined)	0.037
Pain interference		
Carpal tunnel vs. cubital tunnel	50 scored 3, 40 scored 2, 1 scored 0 (carpal tunnel); 2 scored 4, 2 scored 2 (cubital tunnel)	0.38
Carpal tunnel vs. combined decompression	50 scored 3, 40 scored 2, 1 scored 0 (carpal tunnel); all scored 1 (combined)	0.0000366
Cubital tunnel vs. combined decompression	2 scored 4, 2 scored 2 (cubital tunnel); all scored 1 (combined)	0.01
QuickDASH		
Carpal tunnel vs. cubital Tunnel	50 scored 20, 41 scored 10 (carpal tunnel); 3 scored 20, 1 scored 10 (cubital tunnel)	0.438
Carpal tunnel vs. combined decompression	50 scored 20, 41 scored 10 (carpal tunnel); all scored 5 (combined)	0.0000227
Cubital tunnel vs. combined decompression	3 scored 20, 1 scored 10 (cubital tunnel); all scored 5 (combined)	0.009

**Figure 2:**
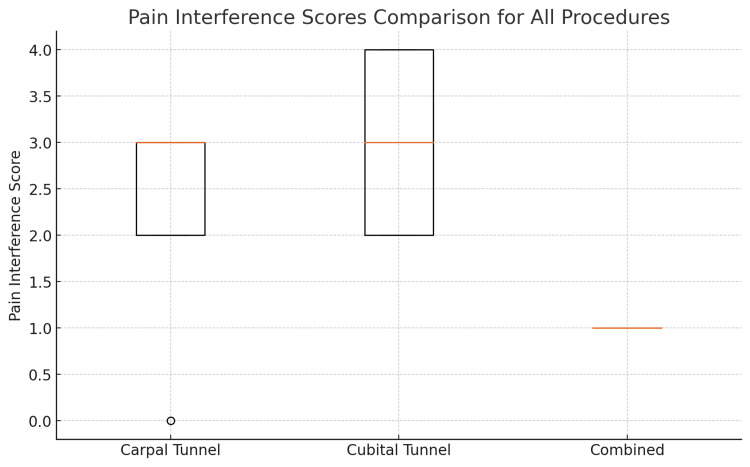
BPI - pain interference This box plot compares pain interference scores among patients who underwent carpal tunnel decompression, cubital tunnel decompression, or both procedures together. The box displays the middle range of scores, with the line inside the box marking the median score. Any scores that are unusually high or low are shown as separate points BPI: Brief Pain Inventory

Pain interference scores were similar between the CTD and CuTD groups, with both reporting a median score of 3/10. However, the CCTD group experienced less interference, with a median score of 1/10. This difference was statistically significant, indicating that patients in the CCTD group had significantly lower pain interference than those in the CTD and CuTD groups (p<0.05) (Figure 2) (Table 1).

**Figure 3:**
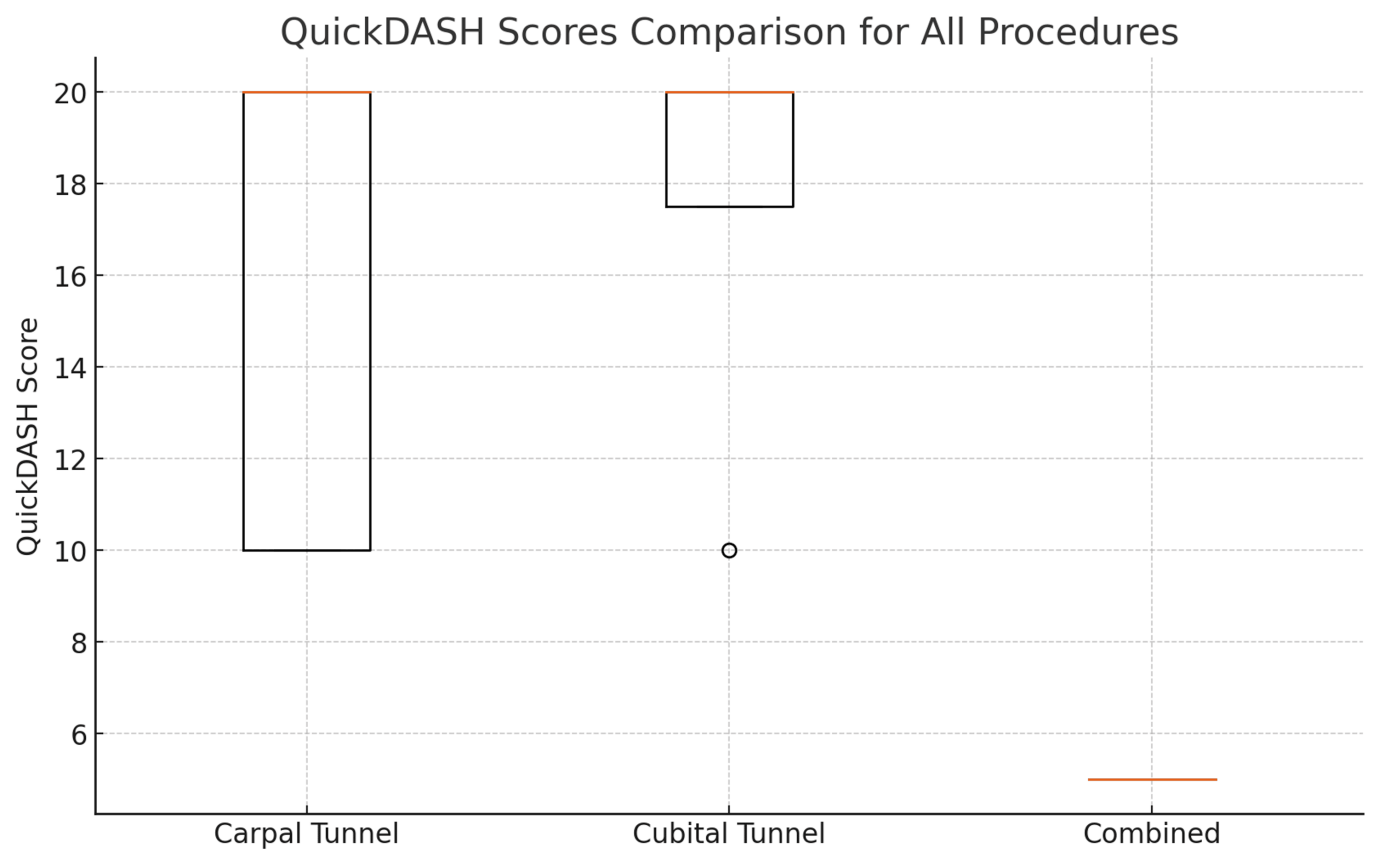
QuickDash This box plot shows a comparison of QuickDASH scores among patients who underwent carpal tunnel decompression, cubital tunnel decompression, or both procedures together. The box displays the middle range of scores, with the line inside the box marking the median score. Any scores that are unusually high or low are shown as separate points QuickDASH: Quick Disabilities of the Arm, Shoulder, and Hand

The QuickDASH score, which measures functional disability, showed that both the CTD and CuTD groups had a median score of 20%. In contrast, the CCTD group reported a much lower median QuickDASH score of 5%, suggesting better functional outcomes. This difference was statistically significant, with the CCTD group showing improved scores compared to the other two groups (p<0.05) (Figure 3) (Table 1).

## Discussion

Numbness, tingling, and pain in the hands are common symptoms that significantly impact the quality of life [1,2]. Key findings from our study indicate that simultaneous surgical decompression of the carpal and cubital tunnels (CCTD) provided greater symptom relief and functional improvement compared to single decompression procedures in patients with concurrent mild to moderate cervical spine stenosis. Patients undergoing combined procedures reported lower pain severity and interference scores, as well as improved QuickDASH scores, indicating the benefits of addressing multiple compression sites simultaneously. The management of hand numbness with concurrent cervical spine stenosis can be challenging. Our study suggests that CCTD may provide more effective symptom relief and functional outcomes than single decompression procedures in such cases. This aligns with previous studies showing that combined surgical interventions can be beneficial, particularly in patients with abnormalities in both the median and ulnar nerves in NCS [11,12].

Our findings further suggest that mild to moderate cervical spine stenosis does not significantly diminish the effectiveness of decompression surgeries for CTS and CuTS, which challenges the "double crush syndrome" hypothesis. This hypothesis states that compression at multiple sites exacerbates symptoms and worsens treatment outcomes. In contrast, our results align with studies reporting improved outcomes for patients undergoing combined decompression when carpal and cubital tunnel compressions coexist with cervical pathology [15,16,18]. The distribution of cervical spine abnormalities within the cohort (67% with mild stenosis, 33% with moderate stenosis) underscores the varied impact of cervical pathology on hand numbness. Future studies with larger, multicenter cohorts could better clarify how different levels of cervical stenosis affect outcomes following decompression surgery.

A major strength of our study was the use of consistent surgical techniques and postoperative care protocols by the same team, minimizing bias and enhancing the accuracy of outcome assessment. However, the study is limited by the small number of patients who underwent simultaneous decompression (n=5), which restricts the generalizability of our findings. While preliminary results suggest combined decompression may be beneficial, these outcomes should be interpreted cautiously, as they provide an initial indication rather than conclusive evidence. Additionally, future research could benefit from including detailed analyses of patient comorbidities and long-term follow-up to evaluate the durability of symptom relief and functional improvement.

Overall, our findings endorse the continued use of surgical decompression for CTS and CuTS in patients with coexisting cervical spine stenosis. This approach may enhance our understanding of the relationship between cervical spine pathology and peripheral nerve compression in the upper extremity.

## Conclusions

Our findings suggest that simultaneous carpal and cubital tunnel decompression is more effective in alleviating symptoms in patients with coexisting mild to moderate cervical spine stenosis. The majority of patients experienced significant symptom relief and improved functional outcomes after surgery. These results emphasize the importance of considering cervical spine pathology when managing hand numbness and highlight the efficacy of decompression procedures in this patient population. However, the small sample size in the subgroup undergoing combined carpal and cubital tunnel decompression (n=5) indicates that these findings are preliminary. Further research with larger cohorts is essential to validate these outcomes and to explore the broader applicability of combined decompression in complex cases involving multiple sites of nerve compression.
